# The application of tissue-engineered fish swim bladder vascular graft

**DOI:** 10.1038/s42003-021-02696-9

**Published:** 2021-10-05

**Authors:** Hualong Bai, Peng Sun, Haoliang Wu, Shunbo Wei, Boao Xie, Wang Wang, Yachen Hou, Jing’an Li, Alan Dardik, Zhuo Li

**Affiliations:** 1 Department of Vascular and Endovascular Surgery, First Affiliated Hospital of Zhengzhou University, Henan, China; 2 Key Vascular Physiology and Applied Research Laboratory of Zhengzhou City, Henan, China; 3 Department of Physiology, Medical school of Zhengzhou University, Henan, China; 4 School of Material Science and Engineering & Henan Key Laboratory of Advanced Magnesium Alloy & Key Laboratory of materials Processing and Mold Technology (Ministry of Education), Zhengzhou University, Henan, China; 5 The Vascular Biology and Therapeutics Program, Yale School of Medicine, New Haven, CT USA; 6 Departments of Surgery and of Cellular and Molecular Physiology, Yale School of Medicine, New Haven, CT USA; 7 Department of Neurology, First Affiliated Hospital of Zhengzhou University, Henan, China

**Keywords:** Physiology, Drug delivery

## Abstract

Small diameter (< 6 mm) prosthetic vascular grafts continue to show very low long-term patency, but bioengineered vascular grafts show promising results in preclinical experiments. To assess a new scaffold source, we tested the use of decellularized fish swim bladder as a vascular patch and tube in rats. Fresh goldfish (*Carassius auratus*) swim bladder was decellularized, coated with rapamycin and then formed into patches or tubes for implantation in vivo. The rapamycin-coated patches showed decreased neointimal thickness in both the aorta and inferior vena cava patch angioplasty models. Rapamycin-coated decellularized swim bladder tubes implanted into the aorta showed decreased neointimal thickness compared to uncoated tubes, as well as fewer macrophages. These data show that the fish swim bladder can be used as a scaffold source for tissue-engineering vascular patches or vessels.

## Abstract (web summary)

Bai et al. employ a fish bladder-derived decellularized matrix for the engineering of vascular grafts. The authors show that rapamycin-coated bladder-derived vascular grafts can be implanted as an interposition graft in rats, and that these vascular grafts showed decreased neointimal thickness both in artery and veins.

## Introduction

Small diameter (< 6 mm) vascular grafts continue to show very low long-term patency after surgery^1^, with > 50% of patients requiring major amputation within a year after failure of an infrageniculate bypass graft^2^. Although polyester and expanded polytetrafluoroethylene vascular grafts have been widely used for more than half a century^3,4^, they do not have acceptable long-term patency and therefore autogenous blood vessels remain the preferred choice of conduit due to their higher patency rate^5^, especially when a ‘no touch’ vein graft harvesting technique is used^6^. Although autogenous vein has satisfactory long-term patency, some patients who need bypass surgery do not have suitable autologous vein; diameter mismatch between the autogenous vein and host vessel remains problematic, and spiral vein grafts are rarely used since they are technically demanding.^7^ Accordingly, several biological vascular grafts have been developed for clinical use, including cryopreserved allografts^8^, human umbilical vein grafts^9^, and xenografts^10,11^. Xenogeneic tissue can be used as a bioengineered vascular scaffold^12,13^, and several commonly used xenogeneic decellularized patches derived from porcine or bovine pericardium are commercially available. Similar to the transition from the first generation of prosthetic materials to the current advanced polymer fabric grafts, the progress in the fields of physiology, cell biology, and biomanufacturing over the past several decades, coupled with recent advances in the tissue-engineering, suggest that next-generation tissue-engineered vascular grafts may become the mainstays of surgical therapy for vascular disease^14^.

Decellularized xenogeneic vascular scaffolds retain the extracellular matrix to maintain the overall shape and architecture of a structure but have fewer immunological reactions due to the decellularization process. Since chitosan extracted from the shells of crab and shrimp appears to be a promising material^15^, we speculated that some organs in bony fish could be similarly processed and used as a scaffold for clinical use. The goldfish *Carassius auratus* is a very common type of fish in China, with high availability. Collagen extracted from fish swim bladders or shark cartilage is similar to mammalian collagen^16–18^; the swim bladder is composed of collagens, elastin, glycosaminoglycan^19^; and may be a potential source for arterial scaffolds. In China, fisheries routinely discard the goldfish swim bladder; each 700–800 g fresh water fish can yield a 5–7 cm swim bladder, suggesting a sustainable source for collagen-based scaffolds.

Fish swim bladder is rarely considered for study in vascular research, with only one reported study; Liu et al.^20^ showed less calcification of fish swim bladder compared with bovine pericardium both in vitro and in vivo, as well as higher patency and lower calcification in a rat arterial model, suggesting that fish swim bladder is a potential candidate cardiovascular biomaterial. However, whether fish swim bladder can be used in the venous system or as a patch, is not known; in addition, it is also not known whether the surface of the fish swim bladder can be coated with a therapeutic drug, and surface modification is currently an important area for research and development^21^. Rapamycin is an effective drug that is widely used to inhibit neointimal hyperplasia both in clinical^22^ and in basic research^23,24^. Previously we showed that pericardial patches can be coated with rapamycin-containing nanoparticles to inhibit neointimal hyperplasia^23^. The decellularized pericardial patch^23^, decellularized human saphenous vein patch^25^, and rat thoracic aorta patch^26,27^ can also be coated with heparin or PD-1 antibody to decrease neointimal thickness. We hypothesized that decellularized fish swim bladder can serve as a biological vascular graft scaffold and can be surface modified; accordingly, we created swim bladder-derived scaffolds, coated them with rapamycin, shaped them into patches or tubes, and implanted them into rats to determine biocompatibility.

## Results

**Fig. 1:**
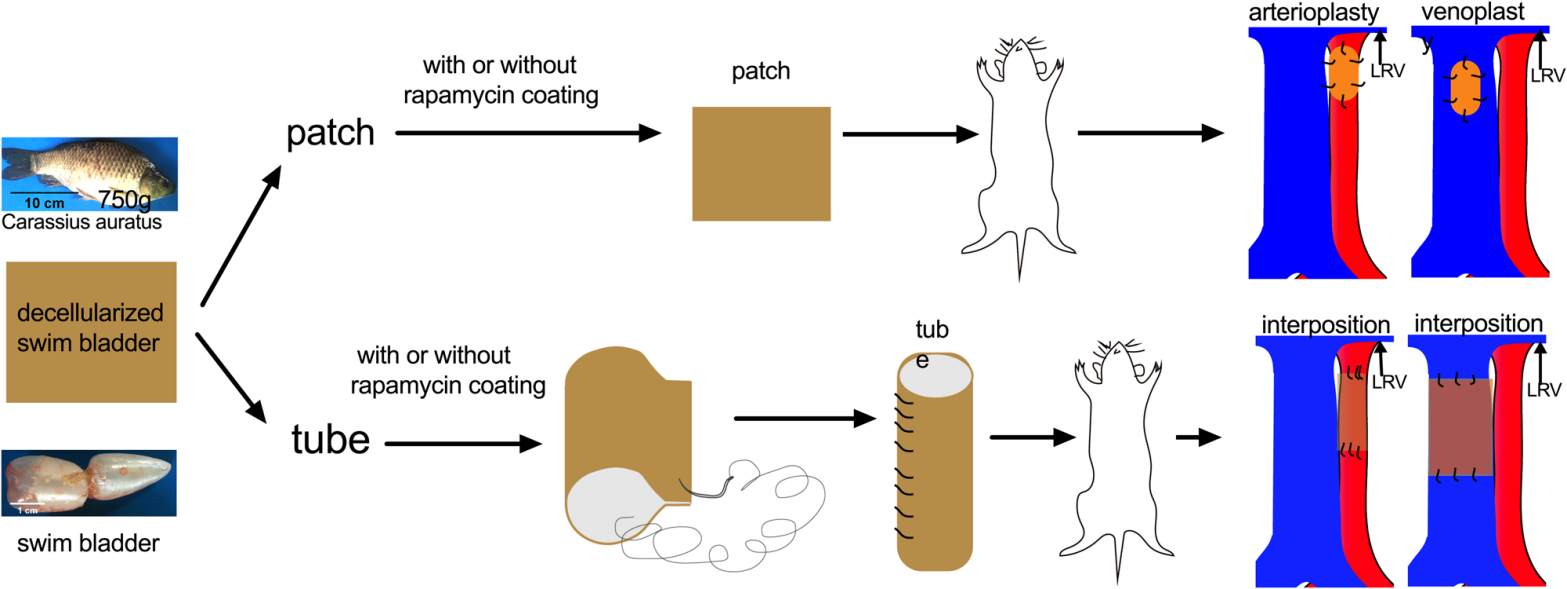
Illustration showing the overall experimental design. LRV, left renal vein. This illustration was created by Dr. Hualong Bai.

**Fig. 2:**
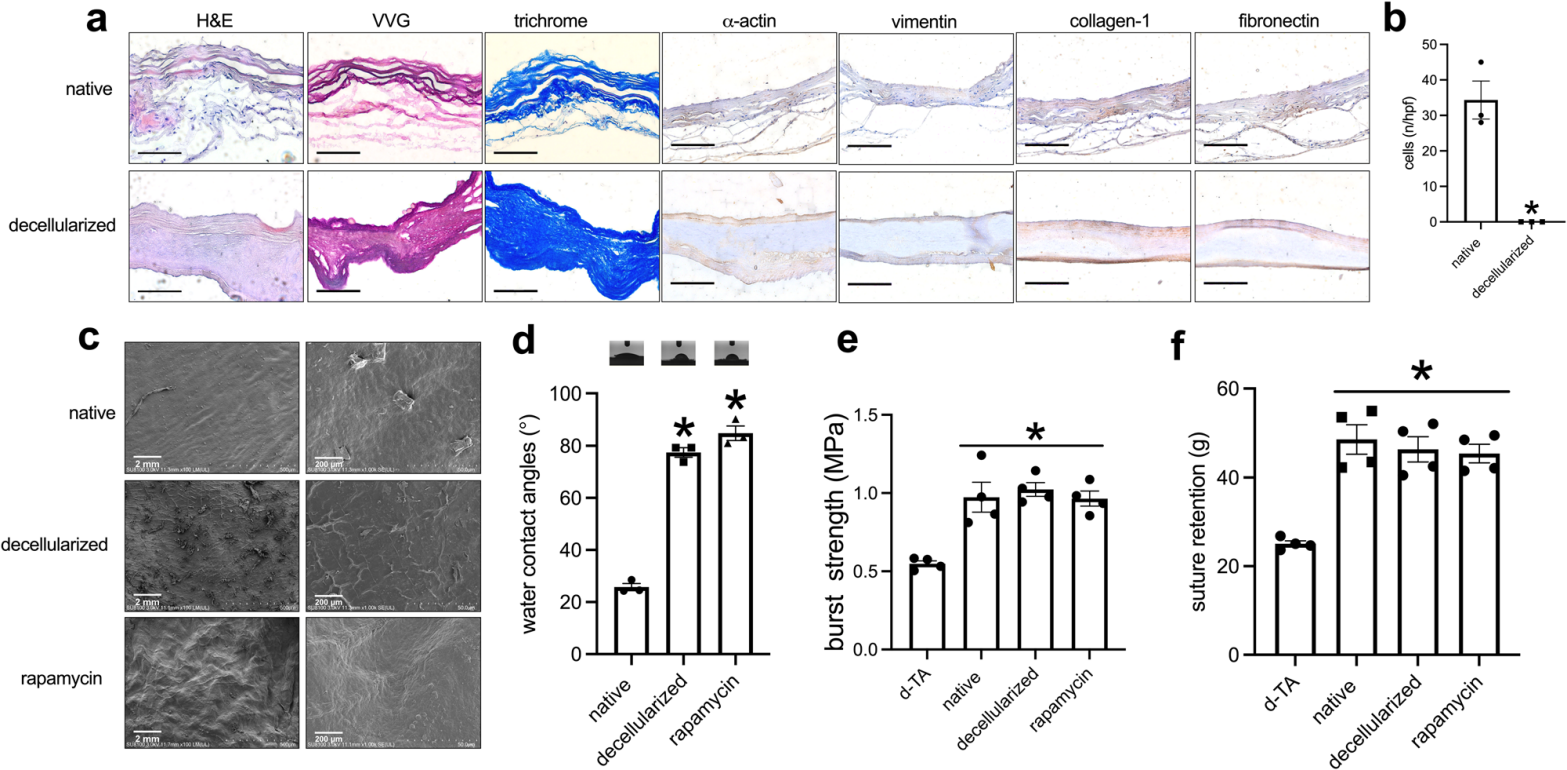
Comparison of the fresh and decellularized fish swim bladder. **a** Photographs showing the swim bladder before and after decellularization, stained with hematoxylin & eosin, VVG, trichrome, α-actin, collagen-1, fibronectin, or vimentin; scale bar, 100 μm; *n* = 3. **b** Bar graph showing the cell number before and after decellularization, *p = 0.0031, *t*-test; *n* = 3. **c** SEM images of fresh, decellularized and rapamycin-coated patches (rapa-coating); scale bar, 200 μm; *n* = 3. **d** Water contact angles of fresh, decellularized and rapamycin-coated patches; the inset panels showing the photographs of the water contact angles; *p* < 0.0001, one-way ANOVA; *p < 0.0001, Tukey’s multiple comparisons test; *n* = 3. **e** Bar graph showing the burst test, *p = 0.0003, one-way ANOVA; d-TA, decellularized rat thoracic aorta; *n* = 4. **f** Bar graph showing the suture retention, *p = <0.0001, one-way ANOVA; d-TA, decellularized rat thoracic aorta; *n* = 4. Data are expressed as mean ± s.e.m.

We assessed the biocompatibility of the fish swim bladder as a source of collagen-based scaffolds (Fig. 1); since the fish swim bladder would be used as a xenograft, to decrease the chance of immunological rejection, we decellularized the swim bladder and examined it, without any additional coating. Hematoxylin and eosin (H&E) staining showed lack of cell nuclei between the wavy collagen fibers (Fig. 2a, b); Verhoeff-Van Gieson (EVG) and trichrome staining, Safranine O, and Sirius Rosa BB staining showed preservation of elastin and collagen fibers after decellularization (Fig. 2a and Supplementary Fig. 1a); Alizarin Red S staining showed no obvious calcification in native and decellularized patches (Supplementary Fig. 1a). Immunofluorescence confirmed lack of any residual cells (Supplementary Fig. 1b), and the DNA content dropped to almost zero after decellularization (Supplementary Fig. 1c). Immunohistochemistry showed α-actin, collagen-1, fibronectin and vimentin were positive stained before decellularization (Fig. 2a); after decellularization there were no α-actin- or vimentin-positive cells, but collagen-1 and fibronectin were preserved. This data shows that decellularization was effective in the fish swim bladder.

To see if the surface of swim bladder could be modified, rapamycin was coated onto the surface of swim bladder. Scanning electron microscopy (SEM) showed a smooth surface before decellularization but a rough surface after decellularization; with rapamycin coating, the surface was a smooth pattern (Fig. 2c). To see the hydrophilic property of surface after modification, water contact angle (WCA) was measured in patches of swim bladder; in the native swim bladder, there was a smaller WCA; after decellularization, there was much larger WCA, and there was no significant difference between the decellularized and rapamycin-coated swim bladder (Fig. 2d). There was a similar burst pressure and suture retention among the native, decellularized and rapamycin-coating patches, with increased burst pressure and suture retention compared to decellularized rat thoracic aorta (d-TA) (Fig. 2e, f). Rapamycin was released for up to 14 days in vitro (Supplementary Fig. 2a). Nanoparticles containing rhodamine (Supplementary Fig. 2b) showed release for up to 24 hours in vivo (Supplementary Fig. 2c). These data show that the decellularized swim bladder can be successfully coated with rapamycin without loss of structural integrity, suggesting suitability for testing in vivo.

We also examined HUVEC apoptosis on the control and hyaluronic acid-rapamycin-coated fish patches. Cells had detectable fluorescence after AO/EB staining, with viable HUVEC showing green color and apoptotic HUVEC showing red color; both the control decellularized fish patches and hyaluronic rapamycin-coated decellularized fish patches showed more viable HUVEC (green) than apoptotic HUVEC (red), and also showed increased numbers of HUVEC over time (Supplementary Fig. 3a). Hyaluronic acid-rapamycin-coated decellularized fish patch had an increased HUVEC vital ratio compared with control decellularized fish swim bladder (Supplementary Fig. 3b). Both patches promoted HUVEC adhesion and proliferation, while hyaluronic acid-rapamycin-coated decellularized fish patches showed better function compared with control patches (Supplementary Fig. 3c, d).

We also examined decellularized fish swim bladder patches with or without rapamycin conjugation implanted subcutaneously or into the abdominal cavity; cells migrated and infiltrated into the patch at days 1, 3 and 7, and the number of infiltrated cells increased over time (Supplementary Fig. 4).

### Patches

**Fig. 3:**
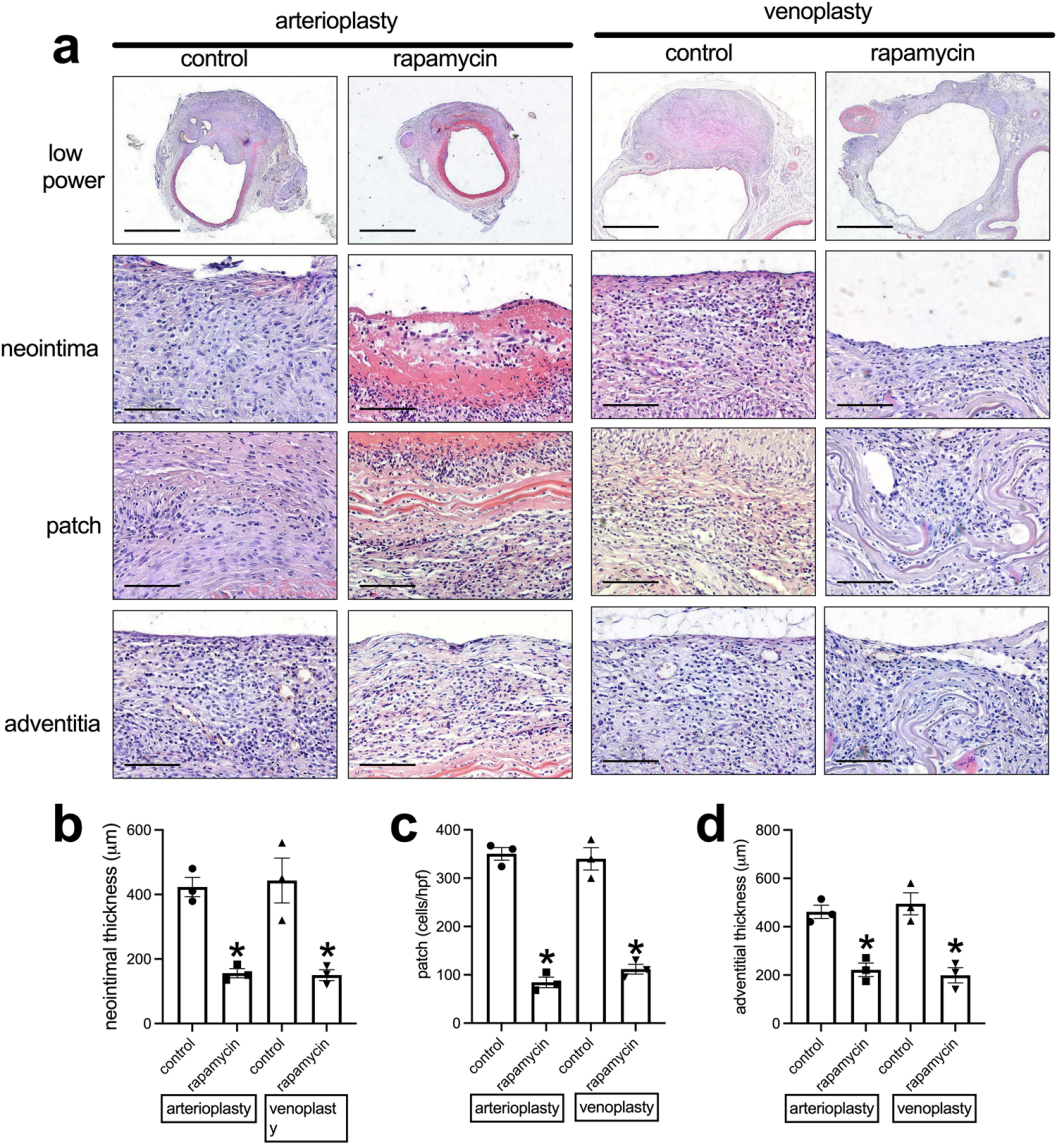
Swim bladder patches harvested from aorta arterioplasty and IVC venoplasty at day 14. **a** Photographs of hematoxylin & eosin staining of the swim bladder patches after arterioplasty or venoplasty, day 14. First row, low power photographs, scale bar, 1 mm; second to fourth rows, high-power photographs shows the neointima, cells infiltrated into the patch and the newly formed adventitia; scale bar, 100 μm; *n* = 3. **b** Bar graph showing the neointimal thickness in the arterioplasty (*p = 0.013) and venoplasty (*p = 0.0147) models, day 14; *t*-test; *n* = 3. **c** Bar graph showing the cells infiltrated into the patch in the arterioplasty (*p = 0.0001) and venoplasty (*p = 0.0008) models, day 14; *t*-test; *n* = 3. **d** Bar graph showing the adventitial thickness in the arterioplasty (*p = 0.0037) and venoplasty (*p = 0.0059) models, day 14; *t*-test; *n* = 3. Data are expressed as mean ± s.e.m.

**Fig. 4:**
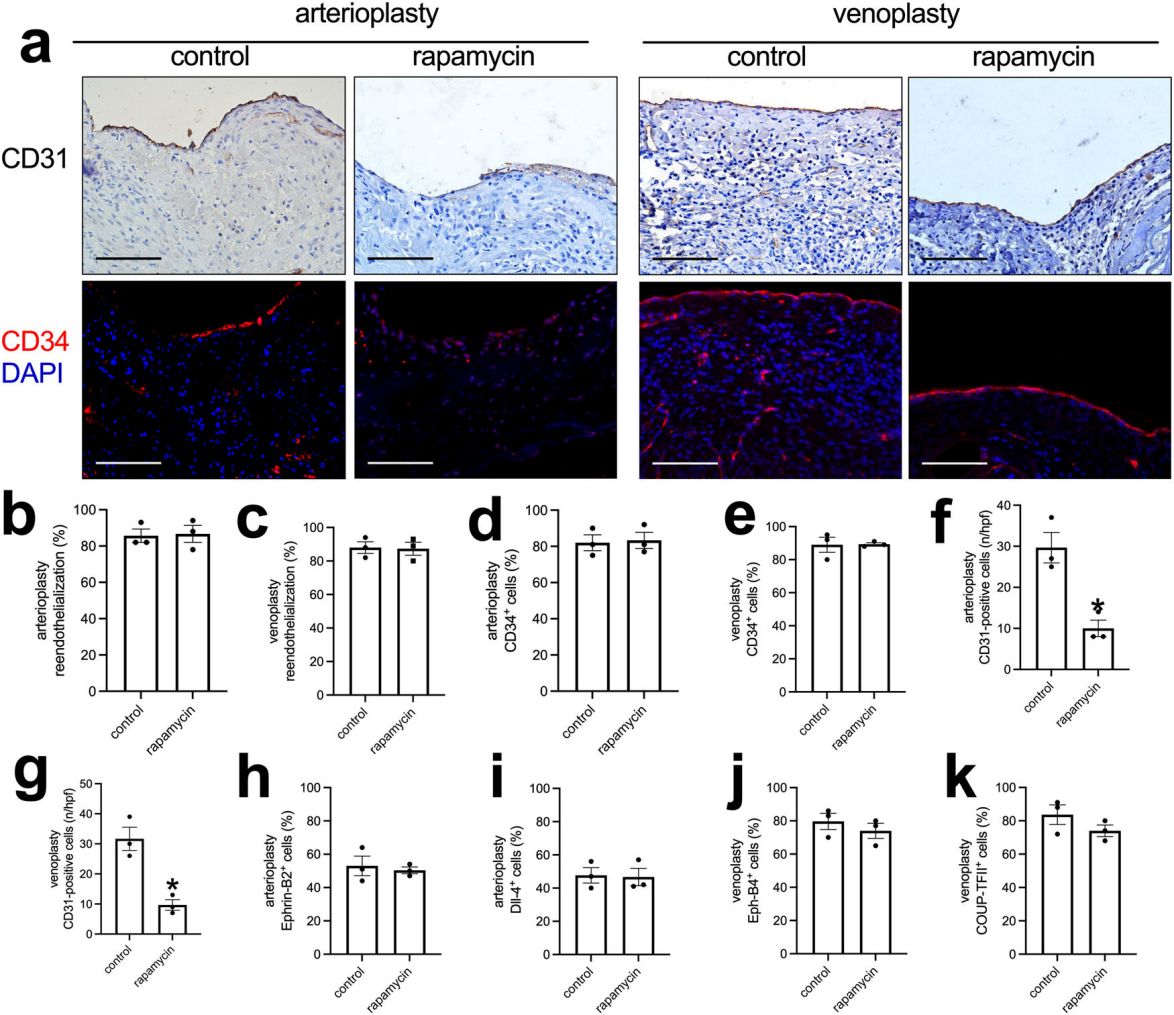
Endothelial cell identity in the control or rapamycin-coated patches harvested from aorta arterioplasty or IVC venoplasty, day 14. **a** First row, Photograph of immunohistochemistry stained for CD31; second row, and photograph of the immunofluorescence merge of CD34 (red) and DAPI (blue) of the neointima; scale bar, 100 μm, *n* = 3. **b**, **c** Bar graphs showing neointimal reendothelialization of the arterioplasty (*p* = 0.8744) and venoplasty (*p* = 0.9037), day 14; *t*-test; *n* = 3. **d**, **e** Bar graphs showing the neointimal CD34-positive cell coverage in the arterioplasty (*p* = 0.8416) and venoplasty (*p* = 0.9465) model at day 14, *t*-test, n = 3. **f**, **g** Bar graphs showing the adventitial CD31-positive cells in the arterioplasty (*p = 0.0096) and venoplasty (*p = 0.0065), day 14; *t*-test; *n* = 3. **h**, **i** Bar graphs showing the percentages of neointimal Ephrin-B2 (*p* = 0.6893) and dll-4 (*p* = 0.8925) positive cells in the arterioplasty, day 14; *t*-test; *n* = 3. **j**, **k** Bar graphs showing the percentages of neointimal Eph-B4 (*p* = 0.4463) and COUP-TFII (*p* = 0.2304) positive cells in the venoplasty, day 14; *t*-test; *n* = 3. Data are expressed as mean ± s.e.m.

**Fig. 5:**
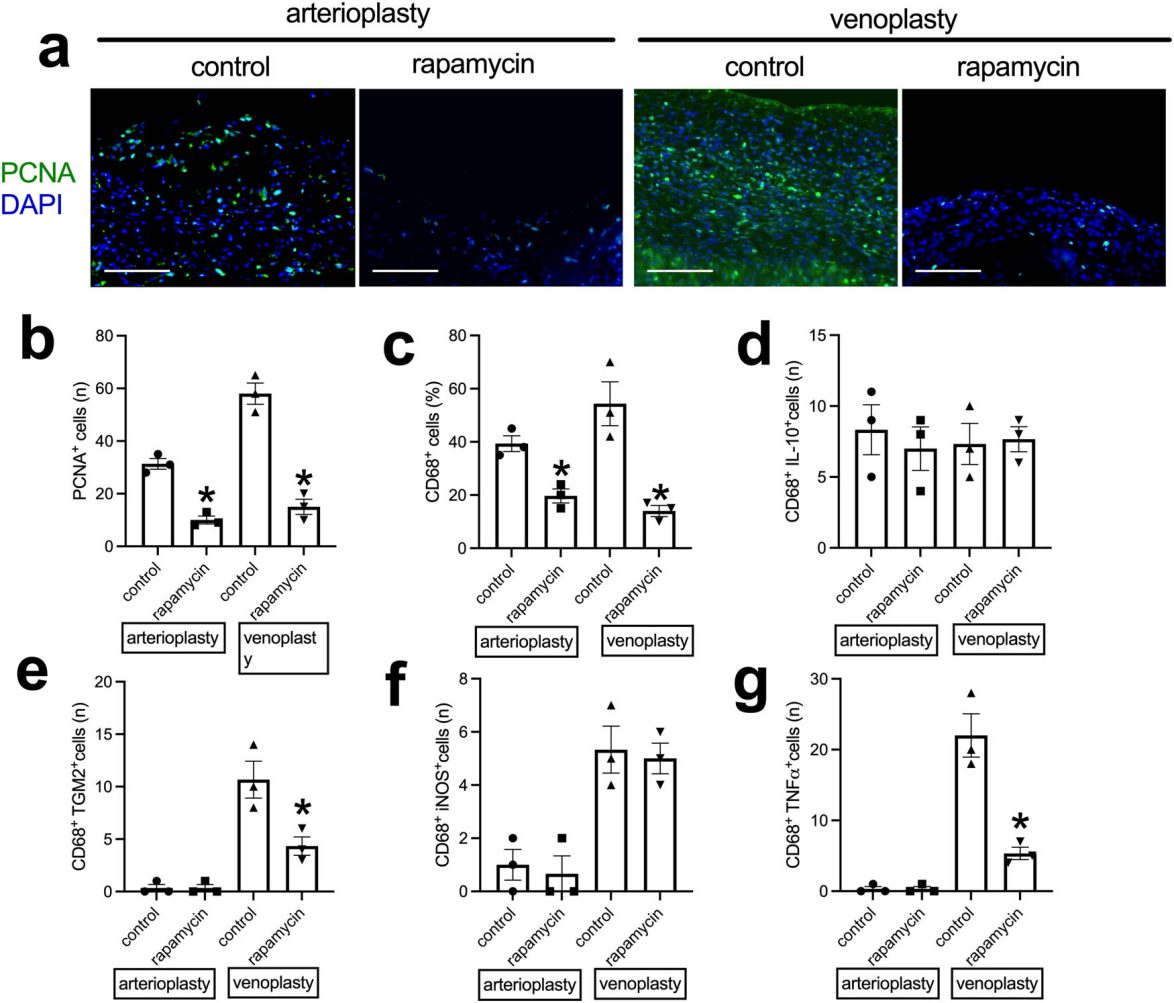
Neointimal cell proliferation and macrophage identity in the control or rapamycin-coated patches harvested from aorta arterioplasty or IVC venoplasty, day 14. **a** Photograph of the immunofluorescence merged of PCNA (green) and DAPI (blue) of the patches; scale bar, 100 μm; *n* = 3. **b** Bar graph showing the neointimal PCNA-positive cells in the control or rapamycin-coated patches in the arterioplasty (*p = 0.0011) or venoplasty (*p = 0.0010) models, day 14; *t*-test; *n* = 3. **c** Bar graph showing the neointimal CD68-positive cells in the control or rapamycin-coated patches of the arterioplasty (*p = 0.0076) or venoplasty (*p = 0.0090) models, day 14; *t*-test; *n* = 3. **d** Bar graph showing the CD68 and IL-10 dual-positive cells in the control or rapamycin-coated patches in the arterioplasty (*p* = 0.5983) or venoplasty (*p* = 0.8541) models, day 14; *t*-test; *n* = 3. **e** Bar graph showing the CD68 and TGM2 dual-positive cells in the control or rapamycin-coated patches in the arterioplasty (*p* > 0.9999) or venoplasty (*p = 0.0325) models, day 14; *t*-test; *n* = 3. **f** Bar graph showing the CD68 and iNOS dual-positive cells in the control or rapamycin-coated patches in the arterioplasty (*p* = 0.7247) or venoplasty (*p* = 0.7676) models, day 14; *t*-test; *n* = 3. **g** Bar graph showing the CD68 and TNF-α dual-positive cells in the control or rapamycin-coated patches in the arterioplasty (*p* > 0.9999) or venoplasty (*p = 0.0063) models, day 14; *t*-test; *n* = 3. Data are expressed as mean ± s.e.m.

We tested the application of the decellularized swim bladder without or with rapamycin coating as patches placed into the rat aorta or the IVC^25,26,28,29^. At day 14, the patches were incorporated into the rat aorta and IVC; there were no aneurysms or occlusions. H&E and EVG staining showed a much thinner neointima in the rapamycin-coated patches compared to the decellularized swim bladder patches (Fig. 3a, b and Supplementary Fig. 5a); immunohistochemistry showed some α-actin-positive cells in the neointima (Supplementary Fig. 5a). There was also less newly formed adventitia on the outer side of the rapamycin-coated patches (Fig. 3a, c), and fewer cells infiltrated into the interspace between the swim bladder fibers in the rapamycin-coated patches (Fig. 3a, d). Immunohistochemistry and immunofluorescence showed a line of CD31 and CD34-positive cells on the luminal surface of the neointima; there was no difference in the amount of reendothelialization or percentage of CD34-positive cells between the control and rapamycin-coated patches (Fig. 4a–e). Within the tissue that formed on the adventitial surface, there were increased numbers of CD31-positive cells, consistent with new capillaries, and there were fewer CD31-positive cells in the rapamycin-coated patches compared to the control patches (Fig. 4f, g and Supplementary Fig. 5b). There were similar percentages of Ephrin-B2 and Dll-4-positive cells on the luminal surface of the neointima in the arterioplasty patches (Fig. 4h, i and Supplementary Fig. 6). There were also similar percentages of Eph-B4 and COUP-TFII-positive cells on the luminal surface of the neointima in the venoplasty patches (Fig. 4j, k and Supplementary Fig. 6), There were fewer PCNA-positive cells in the neointima in the rapamycin-coated patches compared to the control patches in the arterioplasty (Fig. 5a, b) and in the venoplasty (Fig. 5a, b) models. There were fewer CD68-positive cells in the neointima of the rapamycin-coated patches compared to the control patches in the arterioplasty and venoplasty models (Fig. 5c and Supplementary Fig. 7). There were similar numbers of CD68 and IL-10 dual-positive cells, CD68 and TGM2 dual-positive cells, CD68 and iNOS dual-positive cells, and CD68 and TNFα dual-positive cells in the neointima of the rapamycin-coated patches compared to the control patches in the arterioplasty (Fig. 5d–g and Supplementary Fig. 7a). However, in venoplasty, there were similar amount of CD68 and IL-10 dual-positive cells, but fewer CD68 and TGM2 dual-positive cells, similar CD68 and iNOS dual-positive cells, and fewer CD68 and TNFα dual-positive cells in the neointima of the rapamycin-coated patches compared to the control patches (Fig. 5d–g and Supplementary Fig. 7b). These data showed that decellularized swim bladder can be used as a tissue-engineered vascular patch in both the arterial and venous environments and coated with rapamycin to decrease local neointimal thickening.

### Tubes

**Fig. 6:**
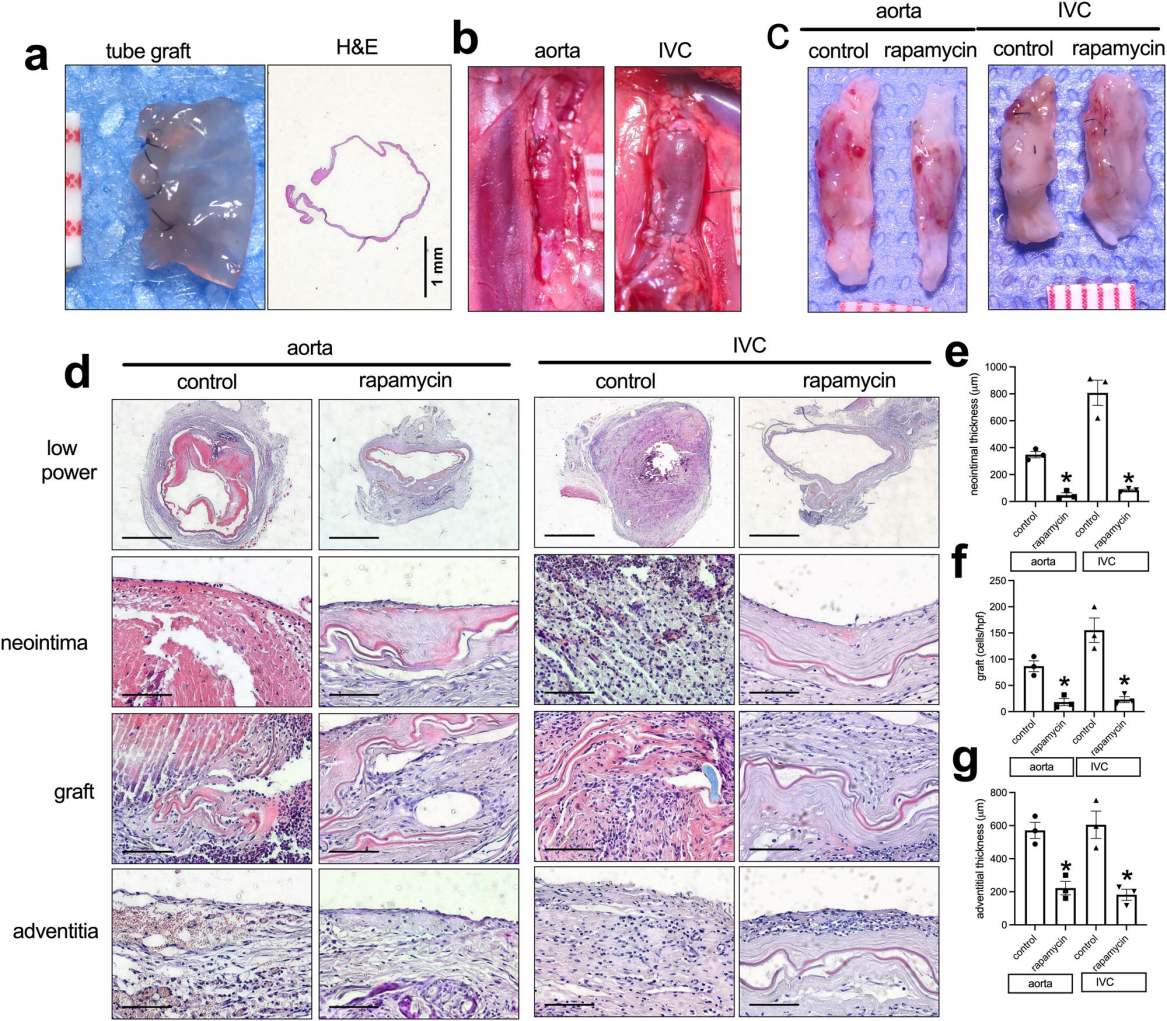
Swim bladder used as a interposition tube graft in the aorta or IVC. **a** Photograph of a vascular tube made of decellularized swim bladder using 11-0 suture; ruler marks 1 mm; H&E staining of a swim bladder tube before implantation. **b** Photographs of swim bladder interposition tube graft in aorta and IVC after completion of the anastomoses; ruler marks 1 mm. **c** Photographs of swim bladder interposition tube grafts, ruler marks 1 mm, day 14. **d** H&E staining of the swim bladder tube grafts, day 14. First row, low power photographs; scale bar, 1 mm. second row, high-power photographs shows the neointima; scale bar, 100 μm; third row, high-power photographs shows the cells infiltrated into the swim bladder; scale bar, 100 μm; fourth row, high-power shows the newly formed adventitia; scale bar, 100 μm. **e** Bar graph showing the neointimal thickness in the aorta (*p = 0.005) and IVC (*p = 0.0015) interposition models, day 14; *t*-test; *n* = 3. **f** Bar graph showing the cells infiltrated into the interposition tube graft in the aorta (*p = 0.0054) and IVC (*p = 0.0092), day 14; *t*-test; *n* = 3. **g** Bar graph showing the adventitia; thickness in the aorta (*p = 0.0055) and IVC (*p = 0.0053) interposition models, day 14; *t*-test; *n* = 3. Data are expressed as mean ± s.e.m.

Since vascular grafts are commonly used for bypass surgeries, we also examined whether swim bladder can be used in a tube configuration. Tubes were made of decellularized bladder by rolling the swim bladder and suturing it with a double layer of sutures (Fig. 6a); tubes of 1 mm diameter were used as aortic interposition grafts and tubes of 2 mm diameter were used as IVC interposition grafts. After implantation, the tube grafts showed good strength without leakage, in both the aorta and IVC (Fig. 6b). After 14 days, all aortic tube grafts were patent; all three IVC tube grafts were occluded in the control group, while the rapamycin-coated tube grafts were all patent (Fig. 6c, d). H&E and EVG staining showed a significantly thinner neointima on the luminal surface of the grafts in the aorta and IVC positions (Fig. 6d, e and Supplementary Fig. 8a). There were also fewer cells infiltrated into the patch of the rapamycin-coated tube grafts compared to the control tube grafts (Fig. 6d, f). There was also a thinner adventitia in the rapamycin-coated tube grafts compared to the control tube grafts (Fig. 6d, g). Immunohistochemistry showed some α-actin-positive cells in the neointima (Supplementary Fig. 8a).

**Fig. 7:**
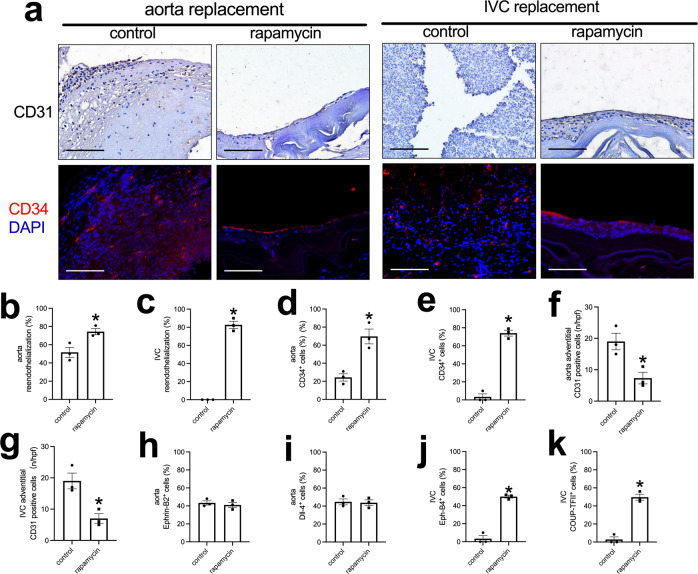
Endothelial cell identity in the control or rapamycin-coated tube grafts harvested from aorta or IVC, day 14. **a** First row, Photograph of immunohistochemistry stained for CD31; second row, and photograph of the immunofluorescence merged of CD34 (red) and DAPI (blue) of the neointima in the tube graft; scale bar, 100 μm, *n* = 3. **b**, **c** Bar graph showing the neointimal reendothelialization of the aortic tube graft (*p = 0.0252) and IVC (*p < 0.0001) tube graft models, day 14; *t*-test; *n* = 3. **d**, **e** Bar graph showing the percentage of CD34-positive cells in the aortic (*p = 0.0077) or IVC (*p = 0.0001) tube grafts, day 14; *t*-test; *n* = 3. **f**, **g** Bar graphs showing the adventitial CD31-positive cells of the aorta (*p = 0.0226) and IVC tube graft (*p = 0.0151) models, day 14; *t*-test; *n* = 3. **h**, **i** Bar graph showing the percentage of neointimal Ephrin-B2 (*p* = 0.6965) and dll-4 (*p* = 0.9404) positive cells in the neointima of the aortic tube grafts, day 14; *t*-test; *n* = 3. **j**, **k** Bar graphs showing the percentage of neointimal Eph-B4 (*p = 0.0002) and COUP-TFII (*p = 0.0004) positive cells in the neointima of the IVC tube grafts, day 14; *t*-test; *n* = 3. Data are expressed as mean ± s.e.m.

**Fig. 8:**
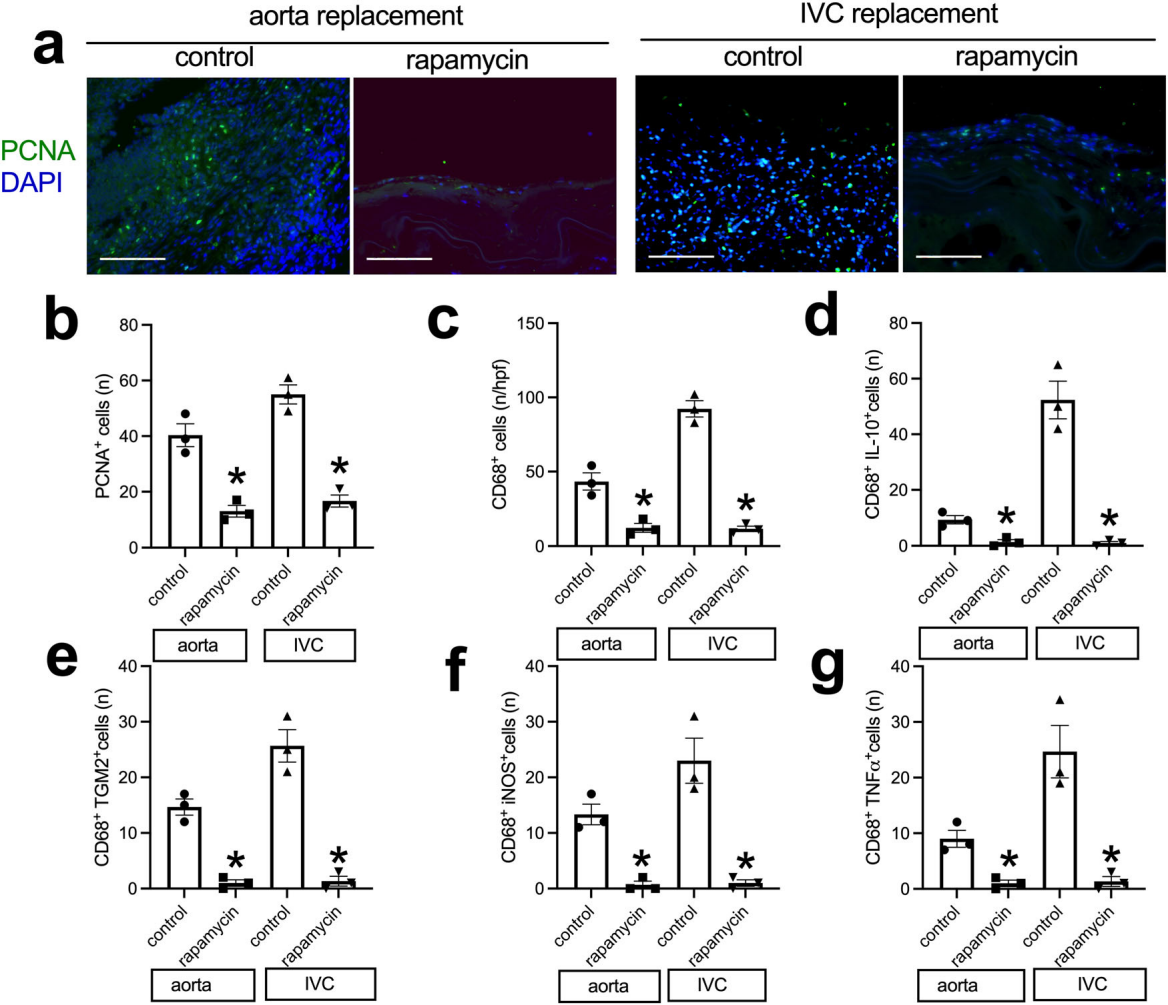
Neointimal cell proliferation and macrophage plastic in the control or rapamycin-coated tube grafts harvested from aorta or IVC, day 14. **a** Photograph of the immunofluorescence merged of PCNA (green) and DAPI (blue) of the neointima in the tube graft; scale bar, 100 μm; *n* = 3. **b** Bar graph showing the PCNA-positive cells of the aortic (*p = 0.0040) or IVC (*p = 0.0007) tube grafts, day 14; *t*-test; *n* = 3. **c** Bar graph showing the neointimal CD68-positive cells in the control or rapamycin-coated tube grafts in the aorta (*p = 0.0090) or IVC (*p = 0.0002), day 14; *t-*test; *n* = 3. **d** Bar graph showing the number of CD68 and IL-10 dual-positive cells in the aortic (*p = 0.0093) or IVC (*p = 0.0016) tube grafts, day 14; *t*-test; *n* = 3. **e** Bar graph showing the number of neointimal CD68 and TGM2 dual-positive cells in the neointima of the aortic (*p = 0.0009) or IVC (*p = 0.0013) tube grafts, day 14. **f** Bar graph showing the number of neointimal CD68 and iNOS dual-positive cells in the neointima of the aortic (*p = 0.0030) or IVC (*p = 0.0057) tube grafts, day 14. **g** Bar graph showing the number of neointimal CD68 and TNF-α dual-positive cells in the neointima of the aortic (*p = 0.0080) or IVC (*p = 0.0082) tube grafts, day 14. Data are expressed as mean ± s.e.m.

Immunohistochemistry and immunofluorescence showed a layer of CD31 and CD34-positive cells on the luminal surface of the neointima except control tube graft in the IVC (Fig. 7a–e). In the adventitial surface of the tube grafts, there were increased numbers of CD31-positive cells, consistent with new capillaries, and there were fewer CD31-positive cells in the rapamycin-coated tube grafts compared to the control tube grafts (Fig. 7f, g and Supplementary Fig. 8b). There were Ephrin-B2 and Dll-4-positive cells on the luminal surface of the neointima on the luminal surface of the aortic tube grafts (Fig. 7h, i and Supplementary Fig. 9); the Ephrin-B2 and dll-4-positive cells on the luminal surface of the neointima on the rapamycin-coated tube grafts appeared more continuous compared to the control grafts (Supplementary Fig. 9). There were more Eph-B4 and COUP-TFII-positive cells in the rapamycin-coated tube grafts compared to the control tube grafts in the IVC (Fig. 7j, k and Supplementary Fig. 9). There were fewer PCNA-positive cells in the neointima of the rapamycin-coated tube grafts compared to the control tube grafts in both the aorta and IVC (Fig. 8a, b). There were fewer CD68-positive cells in the neointima of the rapamycin-coated tube grafts compared to the control tube grafts in the aorta and the IVC (Fig. 8c). There were also fewer CD68 and IL-10 dual-positive cells, fewer CD68 and TGM2 dual-positive cells, fewer CD68 and iNOS dual-positive cells, and fewer CD68 and TNFα dual-positive cells in the neointima of the rapamycin-coated tube grafts compared to the control tube grafts in both the aorta and IVC (Fig. 8d–g and Supplementary Fig. 10a, b). These data show that the decellularized swim bladder can be fashioned as a tube and used in both the arterial and venous environments and coated with rapamycin to decrease local neointimal thickening.

## Discussion

Here, we show that decellularized fish swim bladder can be used as a vascular patch or as an interposition tube graft in both the rat IVC and the aorta, and thus successfully model the patch angioplasty and tube interposition grafts that are surgical procedures commonly used in vascular surgery^7,29^. These decellularized fish swim bladder-based scaffolds can be coated with rapamycin to inhibit both arterial and venous neointimal hyperplasia. These data show that the fish swim bladder can be used as a scaffold source for tissue- engineering vascular patches or vessels.

Tube grafts remain an essential tool for vascular reconstruction. Although native veins are preferred, and prosthetic grafts based on PTFE or polyester are commonly used, there are common clinical situations in which conduits would be preferred, including in the presence of infection or trauma; in addition, large vessels are frequently mismatched in diameter to native vessels such as saphenous vein. We tested the swim bladder in tube configuration in both the rat IVC and aorta; interestingly, all the tubes placed in the IVC were occluded, and all three rapamycin-coated tubes were patent; the lower flow in the venous system and platelet accumulation may have played a role in this process; in addition, the increased proliferation of neointimal cells and/or the larger number of macrophages and M2-type macrophages may have also contributed (Fig. 8). However, swim bladder tubes placed in the aorta were all patent, and there was with a thinner neointima in the grafts coated with rapamycin, suggesting that decellularized fish swim bladder is a potential vascular graft scaffold that can be used for tissue-engineering. Compared to other commercially available prosthetic grafts and biological grafts, the structure of the decellularized fish swim bladder is more similar to native vessels, with increased mechanical strength and stability and excellent hemocompatibility^20^. (Fig. 6).

Neointimal hyperplasia is still a persistent etiology of vascular graft restenosis and failure after vascular interventions^30^, and rapamycin is a commonly used drug that inhibits neointimal hyperplasia in both the clinic as well as research studies^23,24^. We show that decellularized fish swim bladder grafts can be coated with rapamycin and effectively inhibit neointimal hyperplasia in both patch angioplasty and tube interposition graft models (Figs. 3 and 6). Fewer cells infiltrated into the rapamycin-coated grafts, and fewer PCNA and CD68-positive cells were present in the rapamycin-coated grafts, suggesting limited remodeling (Figs. 5 and 8); this limited remodeling of rapamycin-coated grafts may have contributed to increased graft patency, and retention of the native elasticity and compliance. Boada et al.^31^ showed that biomimetic rapamycin nanoparticles can suppress macrophage proliferation and depletion of macrophages could reduce neointimal hyperplasia^32,33^. There were also fewer M2-type macrophages (dual-positive CD68 and IL-10, or dual-positive CD68 and TGM2), fewer M1-type macrophages (dual-positive CD68 and iNOS, or dual-positive CD68 and TNFα) in the neointima of the rapamycin-coated tube grafts compared to the control tube grafts in both the aorta and IVC (Figs. 5 and  8**;** and Supplementary Figs. 7 and 10); this suggests that both M1- and M2-type macrophages may contribute to neointimal hyperplasia in both the arterial and venous environments, which is similar to other studies^34,35^. There were no differences in neointimal reendothelialization, CD34-positive cell number and endothelial cell identity between control grafts and rapamycin-coated grafts, which suggests that rapamycin coated onto fish swim bladder grafts does not influence neointimal reendothelialization in both models, despite some studies that show rapamycin can delay neointimal reendothelization^36,37^. This inconsistency may be secondary to our studies being performed in a small rodent animal model.

There were several limitations in our research. First, we used fish swim bladders derived from fresh water goldfish, which are thinner and smaller than swim bladders derived from salt water fish; thicker swim bladders could give rise to other applications. Second, our research is limited to short time points and longer time points would give more data on longer term performance. Third, we used only a small animal model, and other large animal models that mimic human physiology more closely should also be tested. Fourth, the long-term comparison of fish swim bladder grafts with traditional vascular grafts needs be tested. Fifth, given the high elasticity of the fish swim bladder, the application of fish swim bladder as an arteriovenous graft could also be explored.

We also showed that cells migrated and infiltrated into the decellularized fish swim bladder patch in a process similar to our previous observations that cells infiltrated into pericardial patches^23,38^, polyester patches^29^, decellularized human saphenous vein patches^25^ and decellularized rat thoracic aortic patches^26^. Also similar to other biomaterials, there was rapid reendothelialization after patch implantation or tube interposition; there were no patch pseudoaneurysms after aortic patch angioplasty, suggesting the structural strength and biocompatibility with the arterial environment (Fig. 4). Since the swim bladder-based patches are elastic, there is likely less compliance mismatch compared to prosthetic patches, minimizing anastomotic pseudoaneurysm formation^39^; however, we only observed implantation for 14 days, a short duration of observation, and a longer time of observation is needed to detect longer term compatibility.

The decellularized swim bladder has previously been shown to have anti-calcification properties and excellent hemocompatibility when rolled into multi-layer tubes^20^. Our data confirms these findings and extends these findings to show that surface modification of the scaffold with rapamycin inhibits neointimal formation, a finding of translational significance. Modification of the surface of vascular grafts has showed the ability to inhibit neointimal hyperplasia, and surface modification with heparin^40^ and collagen^41^ have both been used in human vascular surgery. Thus, surface modification of fish swim bladder-based scaffolds is likely to be a promising choice for future applications.

In summary, decellularized fish swim bladder can both be used as patches and tubes in rats, supporting endothelial cell migration and proliferation in vitro and in vivo. The decellularized fish swim bladder is a promising collagen-based scaffold that may be translated to human therapy, including using it for surface coating or modification to inhibit neointimal hyperplasia.

## Methods

The study was approved by the First Affiliated Hospital of Zhengzhou University, Animal Care and Use Committee. All animal care complied with the Guide for the Care and Use of Laboratory Animals. NIH guidelines for the care and use of laboratory animals (NIH Publication #85-23 Rev. 1985) were observed.

### Scaffold decellularization and coating

Fresh water goldfish (*Carassius auratus*) swim bladders were collected from a local market (Zhengzhou city, Henan Province, China). The swim bladder was stored in normal saline containing penicillin (100 U/mL) and streptomycin (100 μg/mL) on ice after harvest and delivered immediately to the laboratory. The fat around the swim bladder can be easily dissected and removed using a dissecting microscope (Nikon, Japan). Decellularization was performed; briefly, the swim bladder was incubated in 250 mL CHAPS buffer (8 Mm CHAPS, 1 M NaCl, and 25 mM EDTA in PBS) for 12 h, followed by a 60 min wash, and then incubated in 10 mL sodium dodecyl sulfate buffer (1.8 mM sodium dodecyl sulfate, 1 M NaCl, and 25 mM EDTA in PBS) for 24 h, followed by wash with PBS to completely remove the detergent^25,42^. Decellularized swim bladder scaffolds were then used for coating or for implantation. The rat thoracic aorta was harvested and decellularized in a similar fashion.

Swim bladders that were coated with rapamycin were immersed into a hyaluronic acid (HA) solution and coated^43^. Briefly, after washing 3 times with phosphate-buffered saline (PBS; 5 min/wash), the HA-coated samples were immersed into a rapamycin solution (2 mg/mL; Zhaoke, Hefei, China) that was also advance-activated in water-soluble carbodiimide solution (15 min), and incubated at 37 ^o^C for 6 h^44^. Rhodamine conjugation was carried out in a similar fashion^26^

### Assessment of rapamycin bonding and release

To show that rapamycin was bonded to the surface of the decellularized swim bladder, we measured the water contact angles (WCA) of the decellularized swim bladder before and after rapamycin bonding, the WCA was measured on the hyaluronic acid rapamycin-coated decellularized swim bladder; morphology was assessed with SEM^45^. Patches conjugated with nanoparticle rapamycin were incubated in PBS at 37 degrees, and the supernatant of each sample was collected and absorption (400 nm) was analyzed at each time point using a SpectraMax plate reader (Molecular Devices, Sunnyvale, CA).

### Suture retention and burst test

Suture retention of the decellularized patches were measured^46^. Briefly, suture retention testing was performed on rectangular specimens (5 cm × 3 cm) clamped at the edges and located opposite to an 8-0 Prolene suture anchored 5 mm from the edge; the suture loop was pulled with a tension meter, and when the suture tore through the patch, the tension was recorded. The burst test measured the pressure at which the swim bladder burst when connected to a calibrated inflation device (BasixCompak, Merit Medical, 30 atm maximum pressure). Uniaxial testing was not performed since it has been previously reported^20^.

### DNA content measurement

DNA extraction from fish swim bladders before and after decellularization was performed using a mouse tail genomic DNA kit (Kangweishiji, Jiangsu, CW2094). Briefly, fresh and decellularized swim bladders were cut into uniformly sized pieces (0.5 × 0.5 cm) and placed in a centrifuge tube. Five-hundred microliters of prepared lysate (5 µL of Proteinase K at 10 mg/mL) was added to each tube, and the tubes were placed in a 55 °C water bath and incubated overnight. The tubes were centrifuged at 13,000 rpm at room temperature for 15 min. Four-hundred microliters of supernatant was pipetted into a new tube, and an equal volume of isopropanol was added and mixed well. The tubes were then centrifuged at 12,000 rpm for 10 min at room temperature. The tubes were air dried on an ultra-clean table for about 3–5 min. The contents were resuspended with 80 µL of pure water and lysed at 55 °C for 2 h; the resuspended liquid was tested using a quantitative DNA (Thermo scientific, Nanodrop 2000) assay.

### Cell culture

Human umbilical vein endothelial cells (HUVEC, ECV304, Oligobio Co.Ltd., Beijing, China) of the 3rd to the 5th passages were seeded on the sterile decellularized fish swim bladder and coated decellularized fish swim bladder (swim bladders were sterilized with 75% alcohol for 12 h) with a density of 1.5 × 10^4^ cells/mL, and cultured in 5% CO_2_ and 37 ^o^C for 4 h or 3 days. Then, the EC were stained with Acridine orange (AO) and Ethidium bromide (EB), and observed under fluorescence microscopy (Ti, Nikon, Japan). The percentage of viable cells (vital ratios) of EC on each sample were counted and calculated from 15 random visual fields. After treating with 4% paraformaldehyde for 1 h at room temperature, the EC were stained with phalloidin and 4,6-diamino-2-phenylindole (DAPI), and the number of EC on each surface were also counted^47^.

### Animal model

Male Sprague Dawley (SD) rats (6–8-week old) were used for all the animal experiments. Anesthesia was given via inhaled isoflurane, and adequate anesthesia was confirmed through a lack of reaction to a toe and tail pinch; ointment on the eyes was placed to prevent dryness while the animals were under anesthesia, and the ventral abdomen hair was removed using a hair remover while wearing sterile gloves. For postoperative analgesia, buprenorphine was given at 0.1 mg/kg intramuscularly no less than every 12 h for 24 h following the surgical procedures. The status of the animal was checked every day in the animal room, ensuring proper recovery from the peri-operative period as well as adequate treatment of post-surgical pain. All the animal models in this research were performed by a skilled vascular surgeon (Dr. Hualong Bai).

The aorta and inferior vena cava patch angioplasty models were performed^28^, microsurgical procedures were performed aseptically using a dissecting microscope (Nikon, Japan). Briefly, the rat abdominal aorta was exposed and dissected free of surrounding structures, and the infrarenal aorta was clamped; a longitudinal 3 mm arteriotomy was then made on the anterior aortic wall, and a control (decellularized but uncoated) or rapamycin-coated swim bladder patch (3 × 1 mm) was sutured in place using running 10-0 nylon sutures. After completion of the angioplasty, the micro clamps were removed and aortic flow was restored. The abdomen was then closed using 5-0 Dacron sutures. The inferior vena cava (IVC) patch venoplasty model was performed using a similar procedure on the IVC (Fig. 1). Rats were sacrificed on postoperative day 14 and the patches were explanted for analysis. To avoid confounding or off-target effects, no immunosuppressive agents, antibiotics, antiplatelet agents, or heparin were given at any time during or after the operative procedure.

The aorta and inferior vena cava interposition models were performed^48,49^. The swim bladder tissue sheets were formed into a tube configuration (inner diameter 1.0 mm for aorta or 2.0 mm for IVC; length 3–5.0 mm; measured directly with a ruler) using 11-0 sutures. The abdominal aorta below the renal artery was exposed and dissected; the proximal and distal aorta were clamped and the aorta transected. The graft was then sutured with an end-to-end configuration using a running 11-0 sutures. The IVC interposition graft was carried out in a similar fashion. Rats were sacrificed on postoperative days 14 and the patches were explanted for analysis; since we found that neointimal hyperplasia was consistent after day 7 in our previous studies^23,29,38^. we chose day 14 as the observational time point^25,26,27^. No immunosuppressive agents, antibiotics, antiplatelet agents, or heparin were given at any time.

At predetermined times the patch and tube grafts were harvested; the rat was anesthetized, the chest was opened, the left ventricle was cannulated with a blunted 20-gauge needle connected to a 20-mL syringe, and an incision was made in the right atrium to allow outflow of perfusion solutions; 100 mL of phosphate-buffered saline (PBS) was infused followed by 10% formalin was perfused by manual pulsatile syringe pressure. The patch or tube graft was carefully removed from the surrounding tissue and stored in 4% neutral buffered formaldehyde.

For the subcutaneous and abdominal cavity implantation models, the control decellularized and rapamycin-coated fish swim bladder patches were implanted subcutaneously and into the abdominal cavity. Patches were harvested at day 1, day 3 or day 7 for analysis.

Nanoparticle-rhodamine (NP-rhodamine) patches were also implanted either subcutaneously or into the abdominal cavity, and then harvested at 6, 12 or 24 hours^23^.

### Histology and EVG staining

Rats were anesthetized with 10% chloral hydrate (intraperitoneal injection), and tissues were fixed by transcardial perfusion of phosphate-buffered saline (PBS) followed by 10% formalin. Tissue was removed and fixed overnight in 10% formalin followed by a 24-h immersion in 70% alcohol. Tissue was then embedded in paraffin and sectioned (4 μm thickness). Tissue sections were deparaffinized and stained with hematoxylin and eosin (Baso, Zhuhai, China), EVG (Baso, Zhuhai, China), trichrome masson (Baso, Zhuhai, China), Alizarin Red S (Solarbio,Beijing,G3280), Safranine O (Solarbio,Beijing,G1371), or Sirius Rosa BB (Solarbio,Beijing,S8060) according to the manufacturer’s recommendations. Neointimal and adventitial thickness were measured as we previously described^38^. The patency of the patch angioplasty and tube graft was confirmed by observation of the histology sections.

### Immunofluorescence

Tissue sections were deparaffinized and then incubated with primary antibodies overnight at 4 °C. The sections were incubated with secondary antibodies for 1 h at room temperature, after which sections were stained with the fluorescent dye 40,6-diamidino-2-phenylindole (Solarbio, Beijing, China) to mark cellular nuclei. Rhodamine conjugated patches were processed as described above and observed directly under the fluorescence microscope.

### Antibodies

Primary antibodies included: anti-CD68 (abcam, ab31360; IF, 1:50); anti-CD31 (R&D, AF3628; IHC, 1:100); anti-α-actin (abcam, ab5694; IF, 1:200); anti-IL-10 (abcam, ab9969; IF, 1:100); anti-iNOS (abcam, ab15323; IF, 1:100); anti-TGM2 (abcam, ab421; IF, 1:100); anti-TNF-α (abcam, ab6671; IF, 1:100); anti-CD34 (abcam, ab81289; IF, 1:50); anti-Ephrin-B2 (Abclone, A5669; IHC, 1:50); anti-Eph-B4 (proteintech, 20883-1-AP; IHC, 1:50); anti-dll4 (Ablcone, A12943; IHC, 1:50); anti-COUP TF II (Abclone, A10251; IHC, 1:50). Secondary antibodies used for IF were from Abclonal, Wuhan, China.

### Statistics and reproducibility

Data are expressed as the mean ± SEM. Statistical significance for these analyses was determined by one-way ANOVA and Tukey’s multiple comparisons test, or *t*-tests (Prism 6; GraphPad Software, La Jolla, CA). *p*-values < 0.05 were considered significant. Animals or independent experiments (*n*) are indicated in the figure legend. Data, expressed as mean ± standard error of mean (s.e.m.), showed high reproducibility between replicate experiments (< %10 variation).

### Reporting summary

Further information on research design is available in the Nature Research Reporting Summary linked to this article.

## Supplementary information

Supplementary Information

Description of Additional Supplementary Files

Supplementary Data

Reporting Summary

## Data Availability

Source data underlying all the main graphs illustrated in this manuscript are available in the Supplementary Data file.
