# Supplementary material for: The application of tissue-engineered fish swim bladder vascular graft
Source: Commun Biol. 2021 Oct 5;4:1153. doi: 10.1038/s42003-021-02696-9 (PMC8492661; doi:10.1038/s42003-021-02696-9)
Supplement: Supplementary file 1 — Supplementary Information [file 42003_2021_2696_MOESM1_ESM.pdf]

The application of tissue-engineered fish swim bladder vascular graft

Hualong Bai[1,3]* Peng Sun[1], Haoliang Wu[1], Shunbo Wei[1], Boao Xie[1],

Wang Wang[2,3], Yachen Hou[4], Jing'an Li[4*], Alan Dardik[5.6]*, Zhuo Li [3,7*]

1. Department of Vascular and Endovascular Surgery, First Affiliated Hospital of Zhengzhou University, Henan, China
2. Department of Physiology, Medical school of Zhengzhou University, Henan, China;
3. Key Vascular Physiology and Applied Research Laboratory of Zhengzhou City, Henan, China
4. School of Material Science and Engineering & Henan Key Laboratory of Advanced Magnesium Alloy & Key Laboratory of materials processing and mold technology (Ministry of Education), Zhengzhou University, Henan, China
5. The Vascular Biology and Therapeutics Program, Yale School of Medicine, New Haven, CT, USA
6. Departments of Surgery and of Cellular and Molecular Physiology, Yale School of Medicine, New Haven, CT, USA
7. Department of Neurology, First Affiliated Hospital of Zhengzhou University, Henan, China

Correspondence: *, Hualong Bai, Department of Vascular and Endovascular Surgery, First Affiliated Hospital of Zhengzhou University, Henan, China, 450052. Tel: +86 18838151596; E-mail: baihualongdoctor@126.com

ORICD ID: https://orcid.org/0000-0003-4039-414X

*, Alan Dardik, Department of Surgery, Yale University School of Medicine, New Haven, CT, US. Fax: +001 203.737.2290; E-mail: alan.dardik@yale.edu

ORICD ID: https://orcid.org/0000-0001-5022-7367

*, Jing'an Li, School of Material Science and Engineering, Zhengzhou University, Henan, China, 450001. Tel: +86 18539956211; E-mail: lijingan@zzu.edu.cn

ORICD ID: https://orcid.org/0000-0002-8311-1739

*, Zhuo Li, Department of Neurology, First Affiliated Hospital of Zhengzhou University, Henan, China, 450052. E-mail: zhuolimarch@126.com

ORICD ID: https://orcid.org/0000-0003-3692-8989

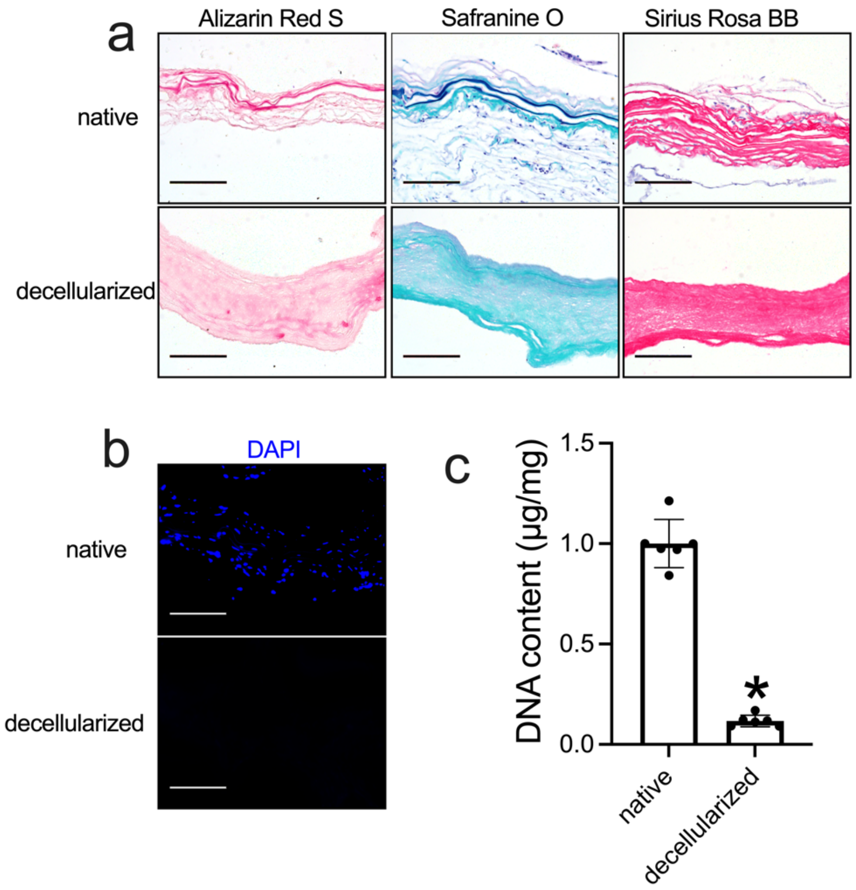

Supplementary Fig. 1. Comparison of native and decellularized fish swim bladder.

a) Photographs showing Alizarin Red S staining, Safranine O staining, Sirius Rosa BB staining; scale bar, 100 µm; n=3. b) Photographs showing fish swim bladder stained with DAPI; there were no residual nuclei in the decellularized fish swim bladder; scale bar, 100 µm; n=3. c) Bar graph showing the DNA content in the native and decellularized fish swim bladder, $p<0.0001$, n=3. Data are expressed as mean± s.e.m.

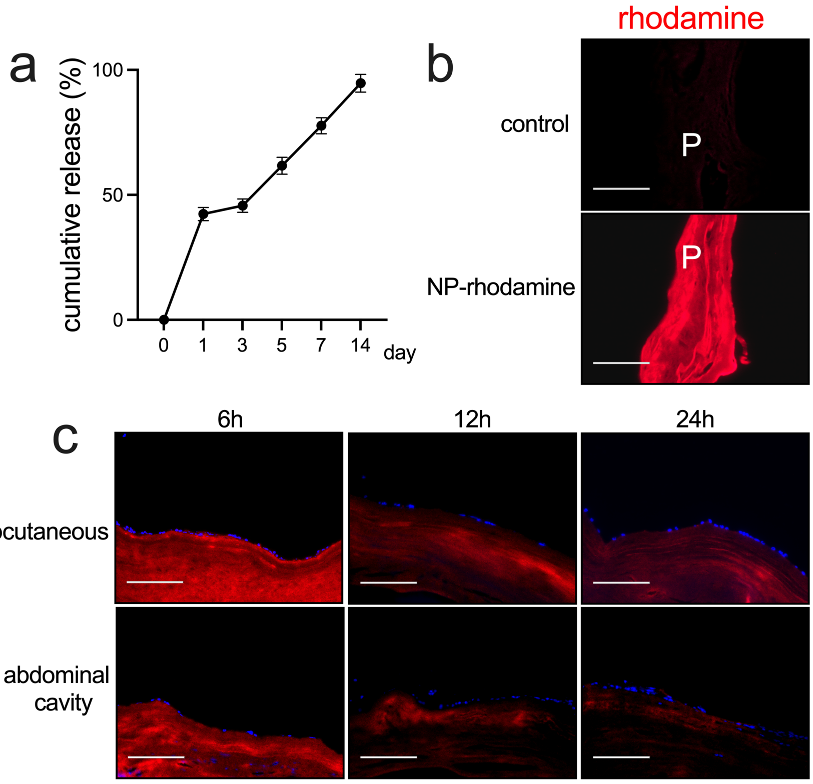

Supplementary Fig. 2. Nanoparticles containing rapamycin or rhodamine conjugated to the decellularized fish swim bladder patch. a) Elution curve showing rapamycin release from nanoparticle rapamycin over 14 days, n=3. b) Patch without and with nanoparticle rhodamine conjugation; scale bar, 100 μm; p, patch; n = 3. c) Time course of nanoparticle rhodamine fluorescence on the patch in the subcutaneous and abdominal implantation at 6h, 12h and 24h after implantation; scale bar, 100 μm; n = 3. Data are expressed as mean± s.e.m.

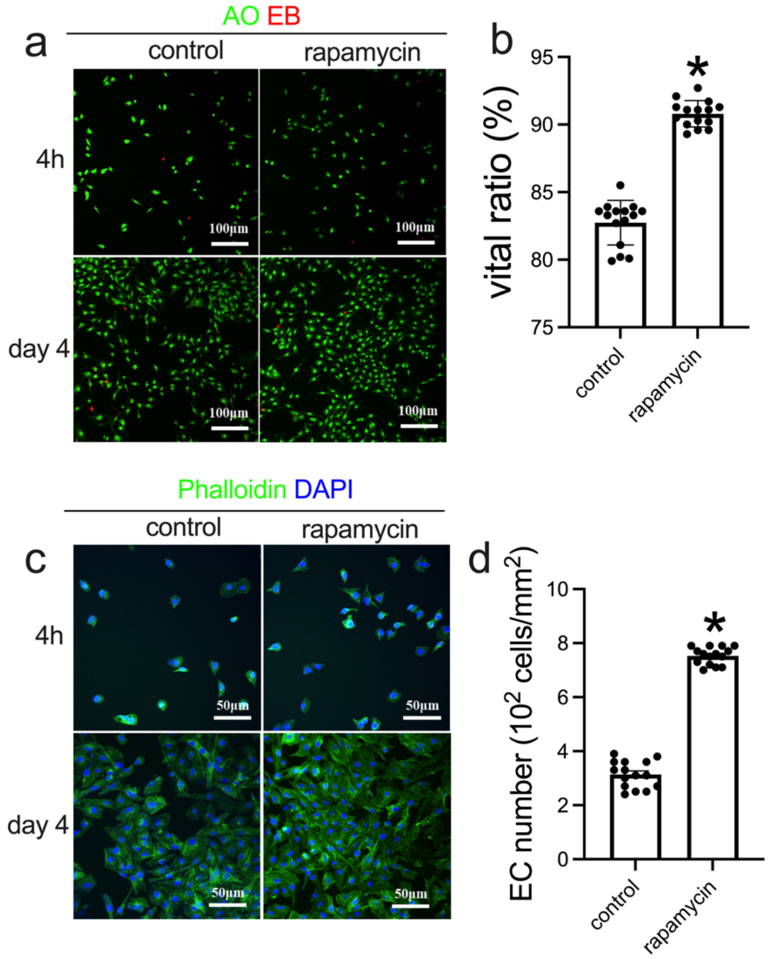

Supplementary Fig. 3. Human umbilical vein endothelial cells (HUVEC) on the control and rapamycin coated patches in vitro. a) Fluorescence images of endothelial cell (EC) stained with AO (Living cells, green) and EB (Apoptotic cells, red) on control patches and rapamycin coated patches, scale bar, 100 μm; n=15. b) Bar graph showing the EC vital ratio on control patches and rapamycin coated patches, *, $p<0.0001$, t-test; n=15. c) Fluorescence images of endothelial cell (EC) stained with phalloidin (green) and DAPI on control patches and rapamycin coated patches (nucleus, blue). d) Bar graph showing EC number on control patches and rapamycin coated patches, *, $p<0.0001$, t-test; n=15. Data are expressed as mean± s.e.m.

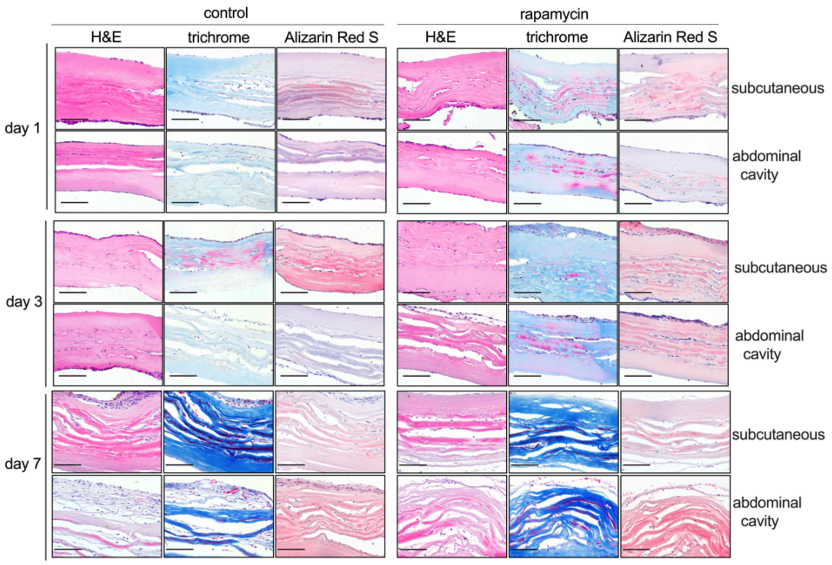

Supplementary Fig. 4. Histology showing the decellularized fish swim bladder with or without rapamycin coating implanted subcutaneously or in the rat abdominal cavity, harvested at day 1, day 3, or day 7; stained with hematoxylin and eosin (H&E), Masson's trichrome, or Alizarin Red S; scale bar, scale bar, 100 μm; n=3.

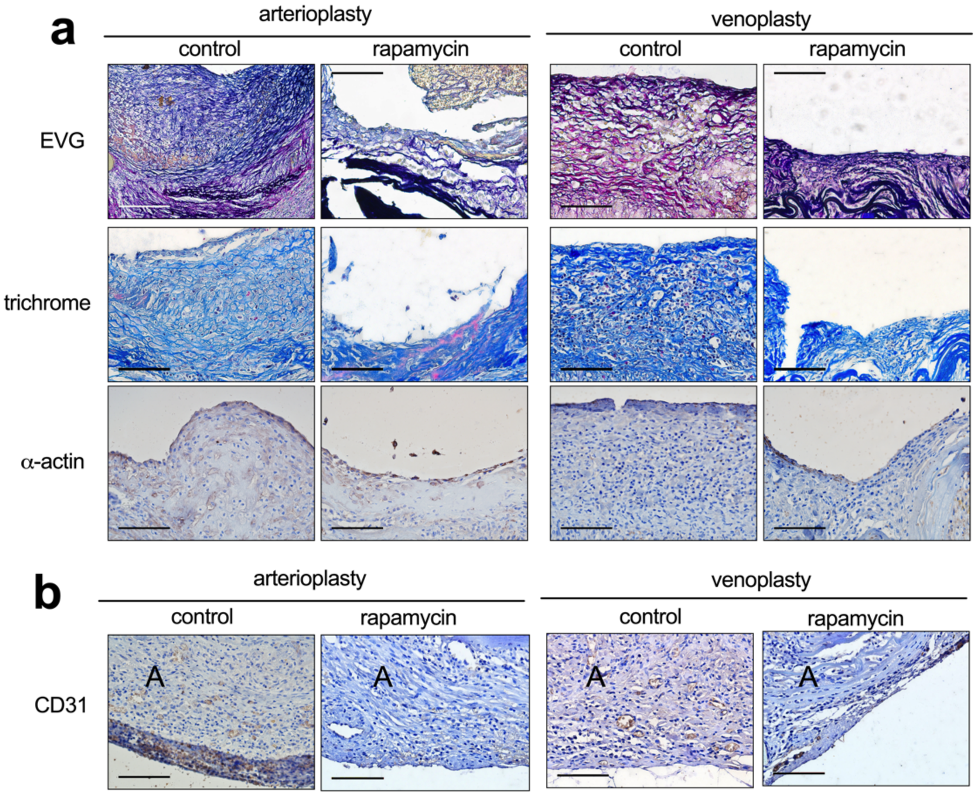

Supplementary Fig. 5. Photographs of the swim bladder control or rapamycin-coating patches, harvested from aorta arterioplasty and IVC venoplasty, day 14. a) First row, high power photographs of the Verhoeff Van Gieson staining showing the neointima; scale bar, 100 μm; second row, high power photographs of the trichrome staining showing the neointima; scale bar, 100 μm; third row, high power photographs showing the neointima stained for α-actin; scale bar, 100 μm; n=3. b) High power photographs showing the adventitia stained for CD31; scale bar, 100 μm; n=3.

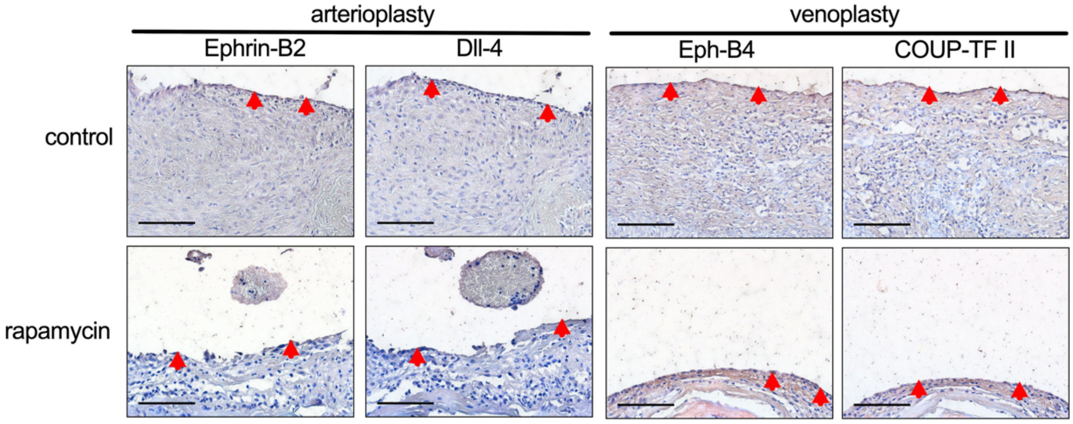

Supplementary Fig. 6. Neointimal endothelial cell identity in the swim bladder, day 14. Immunohistochemistry analysis of the neointima of the patch arterioplasty and venoplasty, day 14. First column, immunohistochemistry stained for Ephrin-B2; second column, immunohistochemistry stained for Dll-4; third column, immunohistochemistry stained for Eph-B4; fourth column, immunohistochemistry stained for COUP-TFII; scale bar, 100 µm; n=3.

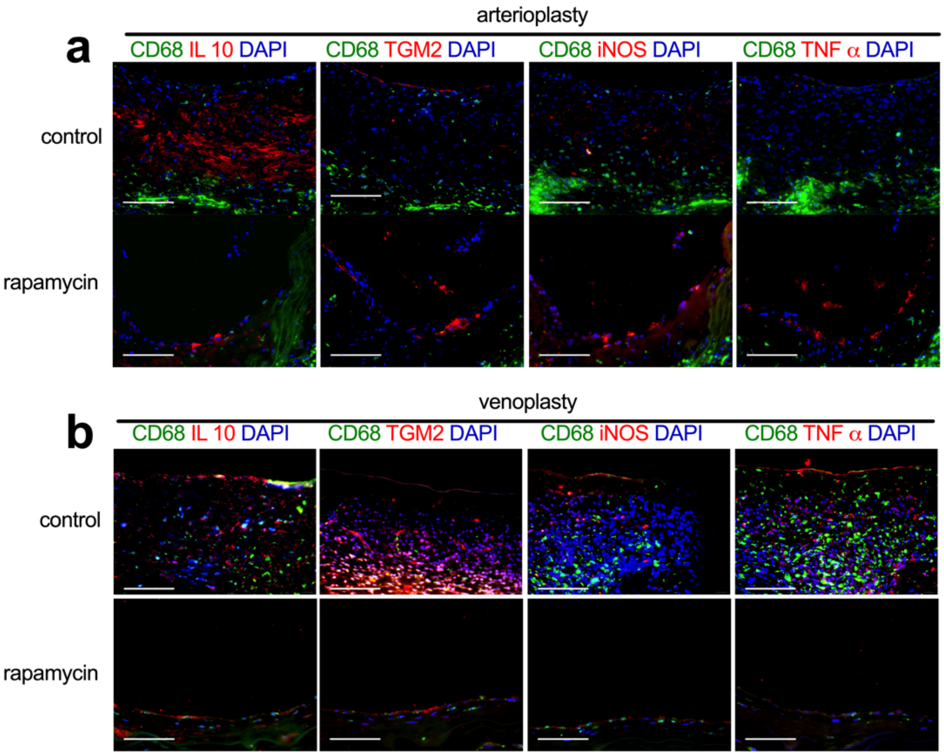

Supplementary Fig. 7. Decreased macrophages in the rapamycin-coated swim bladder patches harvested from aorta arterioplasty or IVC venoplasty, day 14. a) Immunofluorescence analysis of the neointima of the arterioplasty, day 14. First column, merge of CD68 (green), IL10 (red) and DAPI (blue); second column, merge of CD68 (green), TGM2 (red) and DAPI (blue); third column, merge of CD68 (green), iNOS(red) and DAPI (blue); fourth column, merge of CD68 (green), TNFα (red) and DAPI (blue); scale bar, 100 µm; n=3. b) Immunofluorescence analysis of the neointima of the venoplasty, day 14. First column, merge of CD68 (green), IL10 (red) and DAPI (blue); second column, merge of CD68 (green), TGM2 (red) and DAPI (blue); third column, merge of CD68 (green), iNOS(red) and DAPI (blue); fourth column, merge of CD68 (green), TNFα (red) and DAPI (blue); scale bar, 100 µm; n=3.

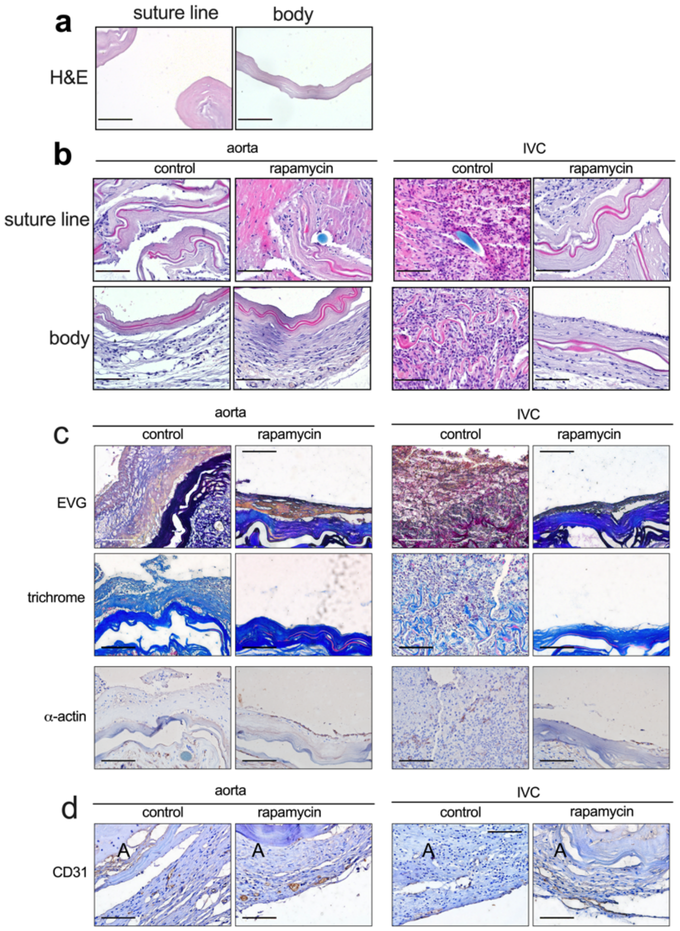

Supplementary Fig. 8. Photographs of the neointima in the control or rapamycin-coated tube grafts harvested from aorta or IVC, day 14. a) Photographs showing the suture line and body (other areas) of the decellularized swim bladder tube, stained with hematoxylin and eosin (H&E), scale bar, 100 µm; n=3. b) High power photographs of the H&E staining showing the suture line and body (other areas) of the tube harvested at day 14; scale bar, 100 µm; n=3. c) First row, high power photographs of the Verhoeff Van Gieson staining showing the neointima; scale bar, 100 µm; second row, high power photographs of the trichrome staining showing the neointima; scale bar, 100 µm; third row, high power immunohistochemistry photographs showing the neointima stained for α-actin; scale bar, 100 µm; n=3. d) High power immunohistochemistry photographs showing the adventitia stained for CD31; scale bar, 100 µm; n=3.

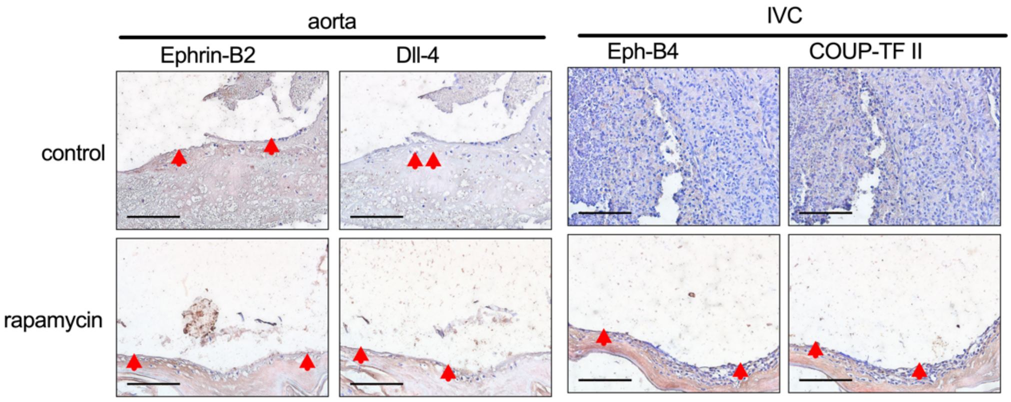

Supplementary Fig. 9. Immunohistochemistry analysis of the neointima of the aorta or IVC interposition tube grafts, day 14. First column, immunohistochemistry stained for Ephrin-B2; second column, immunohistochemistry stained for Dll-4; third column, immunohistochemistry stained for Eph-B4; fourth column, immunohistochemistry stained for COUP-TFII; scale bar, 100 µm; n=3.

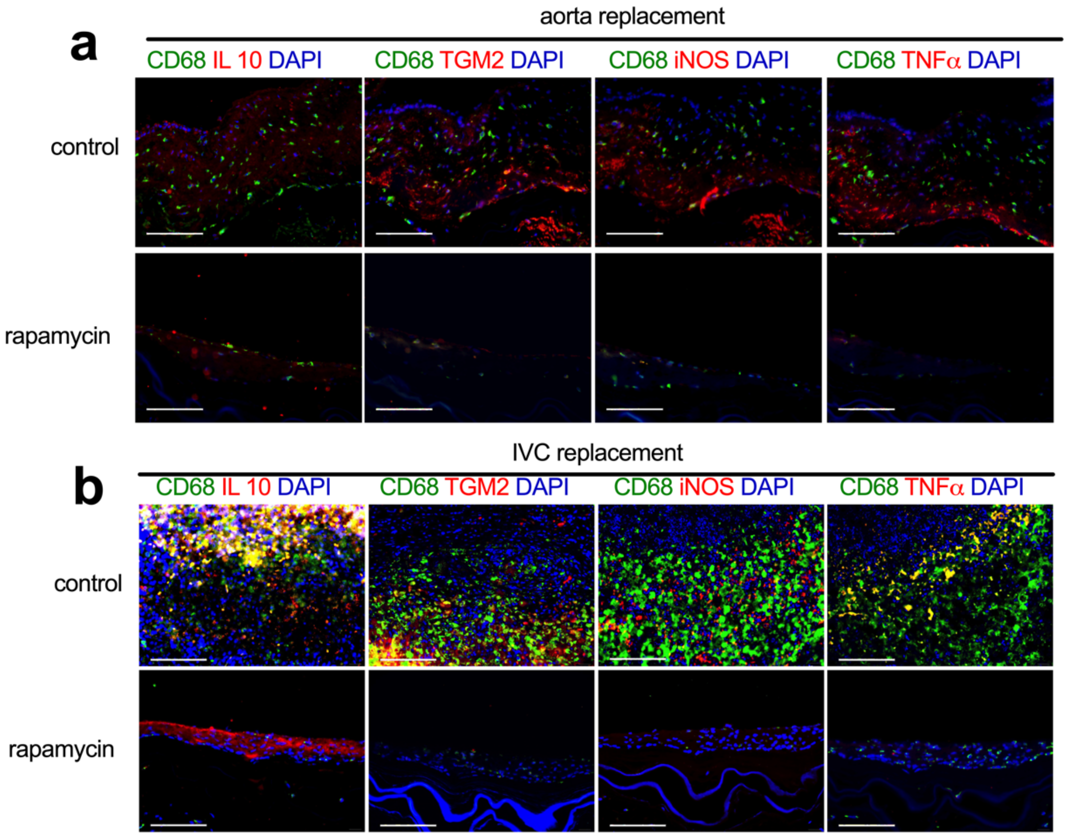

Supplementary Fig. 10. Identity of macrophages in the rapamycin-coated tube grafts, day 14. a) Immunofluorescence analysis of the neointima of the aortic interposition tube graft, day 14. First column, merge of CD68 (green), IL10 (red) and DAPI (blue); second column, merge of CD68 (green), TGM2 (red) and DAPI (blue); third column, merge of CD68 (green), iNOS (red) and DAPI (blue); fourth column, merge of CD68 (green), TNFα (red) and DAPI (blue); scale bar, 100 µm; n=3. b) Immunofluorescence analysis of the neointima of the IVC interposition tube graft, day 14. First column, merge of CD68 (green), IL10 (red) and DAPI (blue); second column, merge of CD68 (green), TGM2 (red) and DAPI (blue); third column, merge of CD68 (green), iNOS (red) and DAPI (blue); fourth column, merge of CD68 (green), TNFα (red) and DAPI (blue); scale bar, 100 µm; n=3.
