# Supplementary material for: The application of tissue-engineered fish swim bladder vascular graft
Source: Commun Biol. 2021 Oct 5;4:1153. doi: 10.1038/s42003-021-02696-9 (PMC8492661; doi:10.1038/s42003-021-02696-9)
Supplement: Supplementary file 2 — Description of Additional Supplementary Files [file 42003_2021_2696_MOESM2_ESM.pdf]

Description of Additional Supplementary Files

File name: Supplementary Data
Description: Source data for Fig. 2-8. The sheets are named according to the figure number.
